# The *Metasequoia* genome and evolutionary relationships among redwoods

**DOI:** 10.1016/j.xplc.2023.100643

**Published:** 2023-06-28

**Authors:** Fangfang Fu, Chi Song, Chengjin Wen, Lulu Yang, Ying Guo, Xiaoming Yang, Ziqiang Shu, Xiaodong Li, Yangfan Feng, Bingshuang Liu, Mingsheng Sun, Yinxiao Zhong, Li Chen, Yan Niu, Jie Chen, Guibin Wang, Tongming Yin, Shilin Chen, Liangjiao Xue, Fuliang Cao

**Affiliations:** 1 State Key Laboratory of Tree Genetics and Breeding, Co-Innovation Center for Sustainable Forestry in Southern China, Nanjing Forestry University, Nanjing 210037, China; 2 Institute of Herbgenomics, Chengdu University of Traditional Chinese Medicine, Chengdu 611137, China; 3 Wuhan Benagen Technology Company Limited, Wuhan 430000, China; 4 Wuhan Botanical Garden, Chinese Academy of Sciences, Wuhan 430074, China; 5 China Academy of Chinese Medical Sciences, Institute of Chinese Materia Medica, Beijing 100070, China

**Keywords:** *Metasequoia glyptostroboides*, redwood, incomplete lineage sorting, polyploidy, flooding stress

## Abstract

Redwood trees (Sequoioideae), including *Metasequoia glyptostroboides* (dawn redwood), *Sequoiadendron giganteum* (giant sequoia), and *Sequoia sempervirens* (coast redwood), are threatened and widely recognized iconic tree species. Genomic resources for redwood trees could provide clues to their evolutionary relationships. Here, we report the 8-Gb reference genome of *M. glyptostroboides* and a comparative analysis with two related species. More than 62% of the *M. glyptostroboides* genome is composed of repetitive sequences. Clade-specific bursts of long terminal repeat retrotransposons may have contributed to genomic differentiation in the three species. The chromosomal synteny between *M. glyptostroboides* and *S. giganteum* is extremely high, whereas there has been significant chromosome reorganization in *S. sempervirens*. Phylogenetic analysis of marker genes indicates that *S. sempervirens* is an autopolyploid, and more than 48% of the gene trees are incongruent with the species tree. Results of multiple analyses suggest that incomplete lineage sorting (ILS) rather than hybridization explains the inconsistent phylogeny, indicating that genetic variation among redwoods may be due to random retention of polymorphisms in ancestral populations. Functional analysis of ortholog groups indicates that gene families of ion channels, tannin biosynthesis enzymes, and transcription factors for meristem maintenance have expanded in *S. giganteum* and *S. sempervirens*, which is consistent with their extreme height. As a wetland-tolerant species, *M. glyptostroboides* shows a transcriptional response to flooding stress that is conserved with that of analyzed angiosperm species. Our study offers insights into redwood evolution and adaptation and provides genomic resources to aid in their conservation and management.

## Teaser

This study reports a chromosome-scale genome assembly for dawn redwood (*Metasequoia glyptostroboides*) and the comparison with those of two other iconic redwood species, *Sequoiadendron giganteum* and *Sequoia sempervirens*. The results suggest that *S. sempervirens* is an autohexaploid and that incomplete lineage sorting explains the inconsistent phylogeny of the redwoods. Transcriptional analysis indicates that the response to flooding stress is conserved between *M. glyptostroboides and* analyzed angiosperms.

## Introduction

The dawn redwood, *Metasequoia glyptostroboides* Hu et Cheng, is the only extant species of *Metasequoia* in the Cupressaceae family. It is well known as a “living fossil” because *Metasequoia* species were known only from fossil records until its discovery in 1943 (Hu and Cheng, 1948; Ma, 2007). The relict *M. glyptostroboides* currently survives only in an enclosed valley along the joint boundary of Hubei and Hunan provinces and in Chongqing municipality in south central China (Hu and Cheng, 1948; Ma, 2007), although *Metasequoia* trees were widely distributed with more morphological variation across the northern hemisphere during the Mesozoic and Cenozoic eras (Yang and Jin, 2000). *M. glyptostroboides* is therefore listed as endangered in the Red List of Threatened Species (www.iucnredlist.org) by the International Union for Conservation of Nature (IUCN).

Since its discovery, many efforts have been made to conserve and distribute *M. glyptostroboides* throughout the world. It currently exists as small populations or solitary trees in more than 50 countries, mainly planted for ornamental purposes (Ma, 2007). The wood of *M. glyptostroboides* has many notable characteristics for construction, furniture, wood fiber (Polman et al., 1999), and raw material for pharmacology (Zeng et al., 2013; Bajpai et al., 2017). Although the number of individuals and the distribution range of *M. glyptostroboides* have increased, the genetic diversity of restored populations is lower than that of natural ones (Li et al., 2003). Low seed germination rates cause difficulties in the natural regeneration of restored populations (Li et al., 2012), and intensive removal of native vegetation has also left fragmented patches of *M. glyptostroboides* in natural sites (Tang et al., 2011).

As early as 1948, Stebbins described the morphological similarity between *M. glyptostroboides* and two redwood species on the west coast of the United States, *Sequoia sempervirens* (coast redwood) and *Sequoiadendron giganteum* (giant sequoia) (Stebbins, 1948). The latter two species are among the most widely recognized and iconic tree species on earth because of their height and volume, and they are also endangered species on the IUCN Red List (Sillett et al., 2015). *S. giganteum* thrives in fragmented groves in the U.S. Sierra Nevada mountain range, and *S. sempervirens* is naturally distributed along the Pacific Coast from southwest Oregon to central California (DeSilva and Dodd, 2020).

The evolutionary relationships among the three extant redwood species have been studied for decades (Schlarbaum et al., 1984). *M. glyptostroboides* and *S. giganteum* are diploids (2n = 2x = 22), whereas *S. sempervirens* is a natural hexaploid (2n = 6x = 66), which is quite rare in gymnosperms (Khoshoo, 1959; Ahuja, 2005; Leitch and Leitch, 2012). On the basis of chromosome configurations, Stebbins suggested that *S. sempervirens* might be an autoallopolyploid produced by hybridization between the ancient *Metasequoia* and some probably extinct taxodiaceous plants (Stebbins, 1948). Seven models for the formation of hexaploid *S. sempervirens* have been proposed through analysis of fossil history, comparative morphology, karyotype analysis, and other data (Ahuja and Neale, 2002). The hybridization process could be involved in formation of an autoallohexaploid (AABBBB or AAAABB), segmental allohexaploid (A1A1A1A1A2A2, A1A1A2A2A2A2, or A1A1A2A2A3A3), or a allohexaploid (AABBCC) (Ahuja and Neale, 2002). Although the distributions of the three modern redwood species are distinct, fossil data suggest that they overlapped across the northern hemisphere from the Cretaceous period (∼145 Mya) (Stockey et al., 2001). On the basis of transcriptome data from the three redwood species, Scott et al. proposed that *S. sempervirens* is most likely an autopolyploid (Scott et al., 2016). Analysis of the *S. sempervirens* genome also indicated that *S. sempervirens* is a partially diploidized autohexaploid (Neale et al., 2021; Ahuja, 2022). The genome sequence of *M. glyptostroboides* and comparative analysis with the genomes of *S. giganteum* and *S. sempervirens* (Scott et al., 2020; Neale et al., 2021) will provide more resources for resolving questions about redwood evolution.

Here, we report a high-quality genome assembly of *M. glyptostroboides* and the analysis of genomic features associated with genome expansion, growth characteristics of giant trees, and conserved responses to flooding stress. Phylogenetic analysis reveals that incomplete lineage sorting (ILS) rather than hybridization has contributed to the genetic polymorphisms and incongruences between gene trees and the species tree of the redwood species. The genome sequence also provides a resource for conservation and management of redwood trees.

## Results

### Chromosome-level assembly of *M. glyptostroboides*

The genome size of *M. glyptostroboides* was estimated to be 7.40 Gb by the K-mer (19-mer frequency) method and flow cytometry (Supplemental Figure 1). Oxford Nanopore Technologies (ONT) sequencing, the Illumina NovaSeq platform, and the Hi-C technique were used to sequence the massive genome of *M. glyptostroboides*. Approximately 430 Gb (58-fold) ONT, 256 Gb (35-fold) Illumina, and 346 Gb (43-fold) Hi-C clean data were generated and used for genome assembly and scaffolding (Supplemental Tables 1 and 2). The genome assembly accounts for 8.07 Gb and contains 1022 contigs with a contig N50 of 12.11 Mb (Table 1). Using the Hi-C data, 99.41% of the contig sequences were anchored onto 11 chromosomes, with chromosome lengths ranging from 452.56 Mb (Chr. 11) to 973.53 Mb (Chr. 3) (Supplemental Tables 3, 4, and Supplemental Figure 1). The quality of the assembled *M. glyptostroboides* genome was evaluated by aligning Illumina short reads to the final assembly, resulting in a high percentage of mapped reads (99.05%) and a large genome coverage (95.07%). Benchmarking Universal Single-Copy Orthologs (BUSCO) analysis revealed that 81.9% of the 1614 core genes were complete (Supplemental Tables 5 and 6). Results from Merqury indicated completeness and base error rates of 97.6% and 0.39%, respectively. These evaluation statistics indicate relatively high completeness of the assembly in gymnosperms.

**Table 1:** Assembly and annotation statistics for the *M. glyptostroboides* genome.

Genome assembly	Number of sequences	Total length, bp	N50, bp	N90, bp	Longest, bp
Contigs	1022	8 074 979 262	12 114 824	3 843 136	62 233 528
Chromosomes	11	8 027 104 625	722 600 678	622 644 211	973 530 065
Unplaced	63	47 979 437	2 038 772	250 000	7 677 550
Final assembly	74	8 075 084 062	722 600 678	622 644 211	973 530 065

Number of genes	Mean gene length, bp	Mean CDS length, bp	Mean exon length, bp	Mean intron length, bp	Number of introns >10 kb
32 184	21 925	1205	360	5207	17 423

### Genome annotation of *M. glyptostroboides*

**Figure 1:**
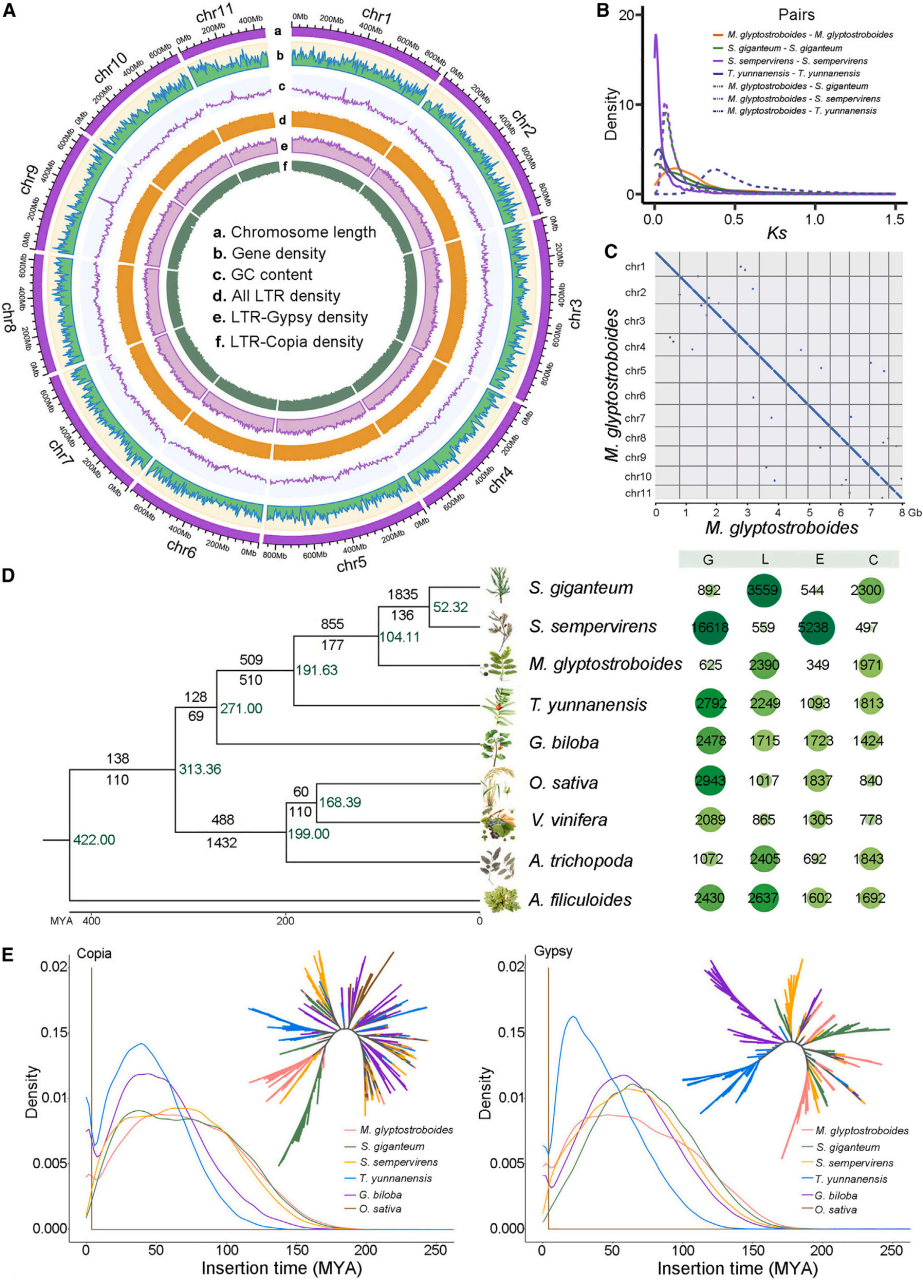
Genomic features of *M. glyptostroboides.* **(A)** Circos plot with different features on the 11 pseudochromosomes. The data were summarized in 10-Mb windows. **(B)** Synonymous substitutions per synonymous site (Ks) distributions of orthologous (and paralogous) genes between *M. glyptostroboides* and *S. sempervirens*, *S. giganteum*, and *T. yunnanensis*. **(C)** Syntenic dot plot of the *M. glyptostroboides* genome. **(D)** Phylogenetic tree of redwoods and selected species and evolutionary analysis of gene families. Numbers next to the branch lines indicate the numbers of expanded (top) and contracted (bottom) gene families. The green numbers next to the nodes represent divergence times. The dot plot on the right shows the gene family numbers of gains (G), losses (L), expansions (E), and contractions (C) in the corresponding species. MYA, million years ago. **(E)** Distributions of insertion times and heuristic maximum likelihood trees of *Copia* (left) and *Gypsy* (right) elements in six plant species. The mutation rates (per base per year) used for calculations were 5.92216e−10 (*M. glyptostroboides*, *S. giganteum*, and *S. sempervirens*), 7.34573e−10 (*T. yunnanensis*), 6.70325e−10 (*G. biloba*), and 1.74e−6 (*O. sativa*). The phylogenetic trees were constructed using amino acid sequences of reverse transcriptase domains.

Repetitive sequences, protein-coding genes, and non-coding RNAs (ncRNAs) were annotated in the genome assembly. Protein-coding gene predictions were obtained from a combination of homology prediction, *ab initio* prediction, and transcriptome assembly models. A total of 32 184 high-quality protein-coding gene models were identified in the *M. glyptostroboides* genome, with average intron and exon lengths of 5207 bp and 360 bp, respectively (Tables 1 and S7). A large proportion of the protein-coding genes (94%) were functionally annotated in various public databases (Supplemental Table 8; Supplemental Figure 2). For ncRNAs, a total of 302 ribosomal RNAs (rRNAs), 6566 transfer RNAs (tRNAs), and 10 729 ncRNAs were predicted (Supplemental Table 9). Repetitive sequences constituted about 63% of the entire genome, about 45% of which were long terminal repeat (LTR) retrotransposons (Figure 1A; Supplemental Table 10).

### Genome duplication analysis of *M. glyptostroboides*

Whole-genome duplication (WGD) is a major driving force during species diversification of flowering plants (Jiao et al., 2011). Analysis of WGD in gymnosperm species can provide information about shared and clade-specific WGD events during gymnosperm evolution. To explore gene duplication patterns, paralogous gene pairs in syntenic blocks were identified in the *M. glyptostroboides genome* and compared with three species in Cupressaceae: giant sequoia (*S. giganteum*), coast redwood (*S. sempervirens*), and *Taxus yunnanensis*. The synonymous substitution rate (Ks) distribution of *M. glyptostroboides* exhibited a single prominent peak at ∼0.12 (range 0–0.5) (Figure 1B), which matches the seed plant WGD event reported in previous studies (Li et al., 2015; Xiong et al., 2021). Intra-genomic collinearity analysis of the *M. glyptostroboides* genome also revealed a lack of extensive syntenic segments (Figure 1C), suggesting that no species-specific WGD occurred during the evolutionary process of *M. glyptostroboides.*

Gene duplication events were annotated and classified in detail on the basis of their chromosomal positions using DupGen_finder (Qiao et al., 2019), which classifies events as transposed duplications (TRDs), proximal duplications (PDs), tandem duplications (TDs), WGDs, and dispersed duplications (DSDs) (Supplemental Figure 3A). DSDs were the most common duplication events identified in the four analyzed species. The number of WGD events was limited in *M. glyptostroboides*, *S. giganteum*, and *T. yunnanensis* compared with *S. sempervirens*. There were 4733, 5446, and 8675 TRD events detected in *M. glyptostroboides*, *S. giganteum*, and *S. sempervirens*, respectively (Supplemental Figure 3A), indicating the significant role of transposons in gene translocation and duplication in the three redwood species. The Ks values of TRD gene pairs were lower in *S. sempervirens* than in the other two related species (Supplemental Figure 3B), suggesting more recent gene translocation in *S. sempervirens*.

### Evolution of gene families in *M. glyptostroboides*

The annotated protein-coding genes were used to study the evolutionary relationships between *M. glyptostroboides* and other gymnosperm species. Molecular phylogenetic analyses using strict single-copy orthologs indicated that *M. glyptostroboides* was clustered with the clades of two sister species, *S. giganteum* and *S. sempervirens* (Figure 1D), consistent with previous studies (Yang et al., 2012; Scott et al., 2016). *M. glyptostroboides* was estimated to have diverged from the *S. giganteum*/*S. sempervirens* clade and *T. yunnanensis* (Cupressaceae Rich. Ex Bartl.) (Song et al., 2021) about 104.1 and 191.6 Mya, respectively. *S. giganteum* and *S. sempervirens* diverged around 52.3 Mya. The results of gene family evolutionary analysis indicated that 625 gene families were exclusive to *M. glyptostroboides*, 2390 gene families had been lost, and 349 and 1971 gene families had undergone expansion and contraction, respectively. The evolutionary patterns of gene families in *S. giganteum* were similar to those in *M. glyptostroboides*, but more gain events of gene families (16 618 genes) were observed in *S. sempervirens* (Figure 1D).

### Clade-specific expansion of retrotransposons in *M. glyptostroboides*

Repetitive sequences, including LTR retrotransposons, made up a large fraction of the *M. glyptostroboides* genome (Supplemental Table 10). Historical TE expansion activity was estimated using the Kimura distance for *Gypsy* and *Copia* LTRs. More than 90% of the *Gypsy* and *Copia* LTR insertions occurred between 8 to 150 Mya. By contrast, all LTR insertions occurred within the past 4 and 8 Mya in rice (Figure 1E). The distribution patterns of insertion times were similar among the three redwood species. The time range of LTR insertions was large for gymnosperms, indicating that LTR insertion was a continuous process in gymnosperms, including *M. glyptostroboides* (Figure 1E)*.* Phylogenetic analysis was performed to explore the evolution of LTRs based on the reverse transcriptase domains of retrotransposons in *M. glyptostroboides* and related species. The results indicated that amplification of *Copia*/*Gypsy* retrotransposons was clade specific in the redwood species (Figure 1E).

### Genome collinearity of three redwood species

**Figure 2:**
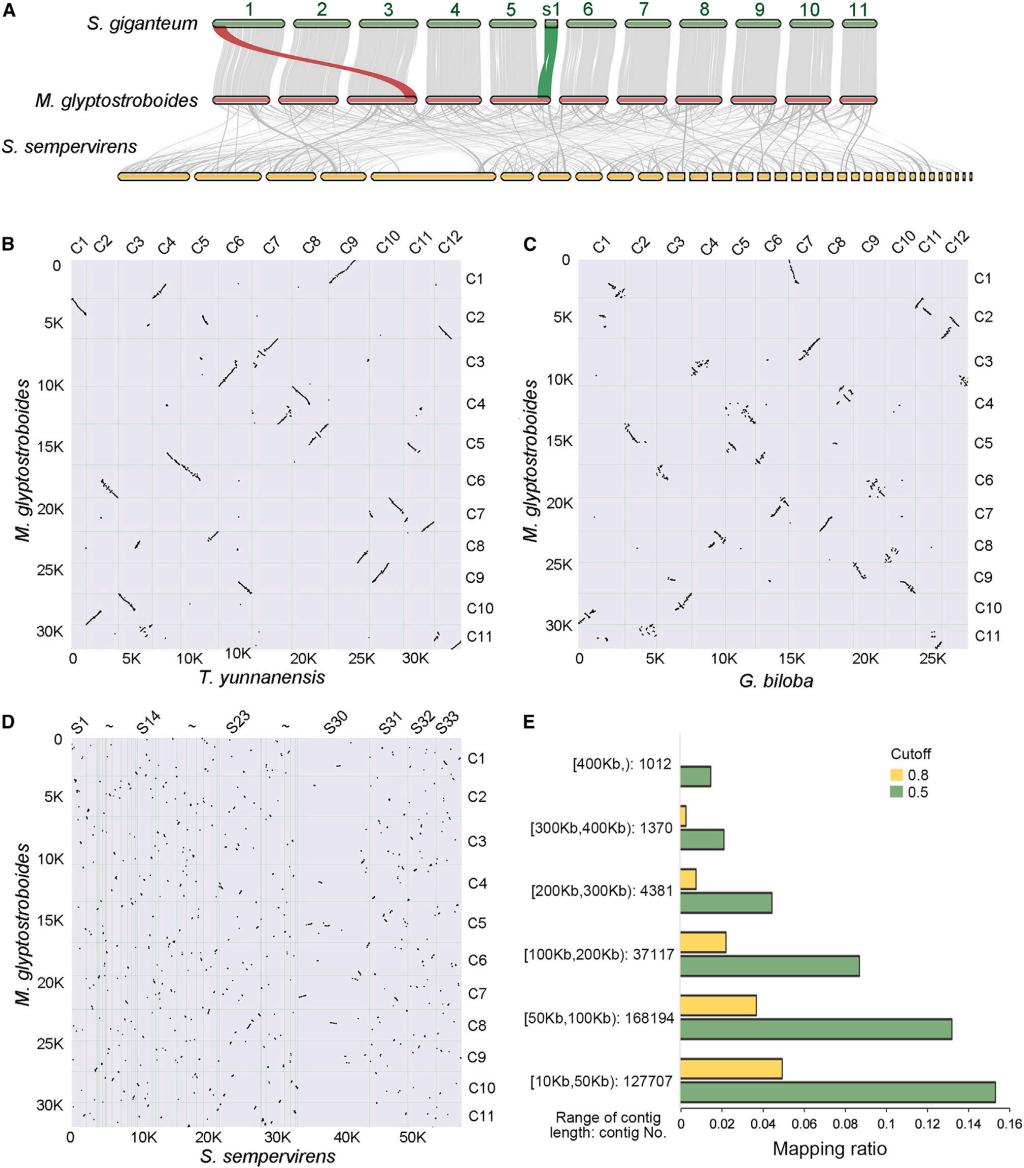
Synteny analysis of genes between *M. glyptostroboides* and other gymnosperm species. **(A)** Syntenic plot of genes between *M. glyptostroboides* and *S. giganteum* and *S. sempervirens*. The gray lines show collinear blocks between species, the red lines indicate the translocation of chromosome fragments between *M. glyptostroboides* and *S. giganteum*, and the green lines indicate collinear blocks between an unanchored scaffold of *S. giganteum* and chromosome five of *M. glyptostroboides*. The genome sequences of *S. giganteum* and *S. sempervirens* were obtained from TreeGenes (https://treegenesdb.org). **(B–D)** Syntenic dot plots of genes between *M. glyptostroboides* and *T. yunnanensis***(B)**, *G. biloba***(C)**, and *S. sempervirens***(D)**. C, chromosomes; S, scaffolds. **(E)** Mapping rate of assembled HiFi contigs from an *S. sempervirens* individual in Nanjing onto the *S. sempervirens* reference genome from the ONT assembly. The contigs were grouped by length and mapped with cutoffs of 0.8 and 0.5, respectively. The ranges are shown in half-open intervals to indicate that the smaller numbers are included.

Chromosomal rearrangements reduce gene flow and play a significant role in speciation events (Rieseberg, 2001). Inter-species genome collinearity was analyzed to explore the degree of chromosomal rearrangement among redwood species. The genomes of *S. giganteum* and *S. sempervirens* were sequenced and assembled previously using a combination of deep-coverage Illumina sequencing, long-read ONT sequencing, and chromosome conformation capture libraries. The genome of *S. giganteum* was assembled at the chromosome-scale scaffold level (Scott et al., 2020) and that of *S. sempervirens* at the partial chromosome-arm level (Neale et al., 2021). The *M. glyptostroboides* and *S. giganteum* genomes displayed an extremely high degree of collinearity, with almost one-to-one chromosome correspondence except for one translocation and one unanchored scaffold in *S. giganteum* (Figure 2A). In total, 14 132 gene pairs in 94 blocks (150.3 gene pairs per block) were identified between *M. glyptostroboides* and *S. giganteum* (Supplemental Table 11)*.*

By contrast, the genome collinearity between *M. glyptostroboides* and *S. sempervirens* was very low (13.3 gene pairs per block), even lower than that of *M. glyptostroboides* and ginkgo (14.7 gene pairs per block) (Figures 2B–2D; Supplemental Table 11). The significant genomic divergence between *S. sempervirens* and *M. glyptostroboides*/*S. giganteum* indicated substantial chromosomal rearrangement during the evolution of *S. sempervirens.* The number of paralogous syntenic blocks in *S. sempervirens* was very low (Supplemental Figure 4), indicating that major chromosomal rearrangement had occurred after polyploidization. To evaluate how chromosome rearrangement affected the chromosomes of *S. sempervirens* individuals, we resequenced the genome of one *S. sempervirens* plant using the HiFi technique. High-quality reads were assembled into a 23.3-Gb draft genome with an N50 of 71.8 kb. More than 74% of the raw HiFi reads could be mapped onto the assembly at a cutoff of 0.80 in total length. The alignment between the draft HiFi assembly and the reference ONT assembly indicated substantial divergence between the two individuals, despite the fact that they were from the same species. Of the 1012 HiFi contigs longer than 400 kb, none could be mapped onto the ONT reference at a 0.8 cutoff of total length, and only 1.5% could be mapped at a 0.5 cutoff. About 15% of the HiFi contigs with lengths 10–50 kb could be mapped at the 0.5 cutoff (Figure 2E).

### ILS in redwood species

**Figure 3:**
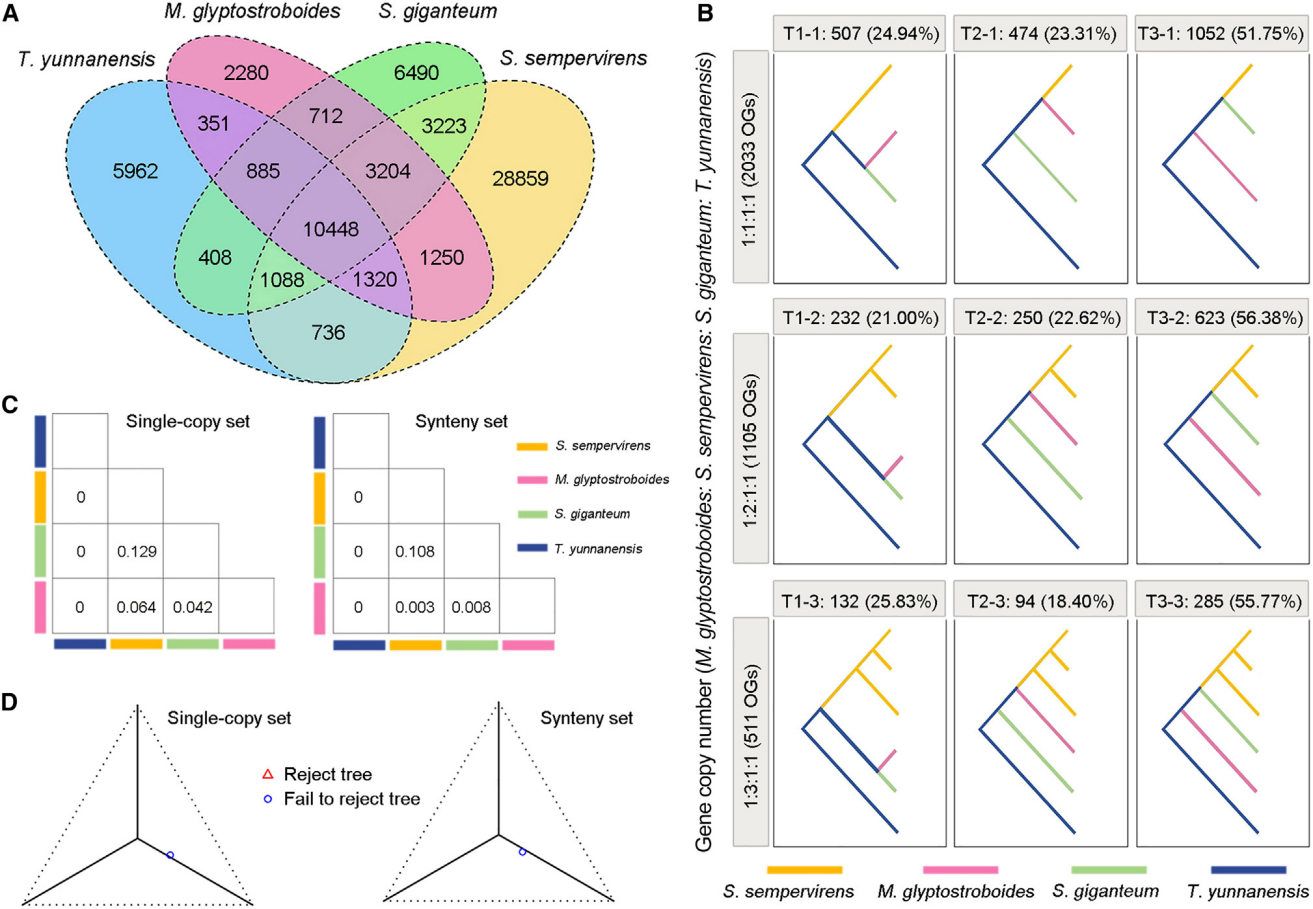
Phylogenetic relationships among three redwood species. **(A)** Venn diagram showing shared and unique gene families in four species. **(B)** Nine major phylogenetic topologies of orthologous genes in the redwoods. The pattern numbers indicate the gene copy numbers in each ortholog group in the order of *M. glyptostroboides*: *S. sempervirens*: *S. giganteum*: *T. yunnanensis*. T1–T3 represent three alternative phylogenetic relationships among the redwoods. **(C)** The mean total proportion of introgressed gene loci per species pair inferred by QuIBL analysis for single-copy (left) and syntenic (right) gene sets. **(D)** Simplex plots of quartet concordance factors (qcCFs) from MSCquartets for single-copy (left) and syntenic (right) gene sets. The multi-species coalescent (MSC) model of ILS and the T3 model with no specific species-tree topology were used for the analysis.

The whole-genome sequences of *M. glyptostroboides* and the other two redwoods provide valuable resources for evaluating their evolutionary relationships. When orthologous groups (OGs) were identified for the three redwood species and *T. yunnanensis*, a large proportion were shared by the four species, and about 29 000 OGs (containing 38 038 genes) were specific to *S. sempervirens* (Figure 3A)*.* Among the OGs specifically present in *S. sempervirens*, there were twice as many OGs with two gene copies than with three gene copies. Among the OGs shared by the three redwood species, OGs with a single gene copy in *S. sempervirens* were most numerous (Supplemental Figure 5).

We next focused on OGs with single genes in *M. glyptostroboides*, *S. giganteum*, and the outgroup *T. yunnanensis*. Among these OGs, 2258, 1596, and 958 contained one, two, and three gene copies, respectively, in *S. sempervirens*. The number of OGs with three gene copies in *S. sempervirens* was much smaller than that of OGs with one gene copy in *S. sempervirens* (Supplemental Table 12). The CDSs of genes in each of these OGs were selected, concatenated, and aligned for phylogenetic analysis. The results from OGs with high-confidence bootstrap support values (≥50) indicated that the phylogenetic trees could be grouped into nine major topologies (Figure 3B). Among these topologies, the duplicated genes (1105 OGs with two copies and 511 OGs with three copies) in *S. sempervirens* were clustered together, indicating that *S. sempervirens* is an autopolyploid.

For 2033 OGs with one copy in *S. sempervirens* and high-confidence bootstrap support values, about 51.75% of the trees were consistent with the species tree that showed the *S. sempervirens*–*S. giganteum* topology, whereas 24.94% and 23.31% were consistent with the *M. glyptostroboides*–*S. giganteum* and *S. sempervirens*–*M. glyptostroboides* topologies (Figure 3B). These inconsistent phylogenies could be caused by ILS (Scott et al., 2016) and introgression or hybridization. We used different software tools to estimate the effects of these two mechanisms using the single-copy gene set. The results from QuIBL indicated that the total proportion of introgressed gene loci was very low, which did not support introgression as the major reason for the inconsistent phylogeny (Figure 3C; Supplemental Table 13). We next used MSCquartets to infer species networks under the multi-species coalescent (MSC) model. The statistical results indicated that two alternative minor topologies had similar concordance factors (CFs), providing support for acceptance of the H0 hypothesis: ILS makes the main contribution to the discordant topologies (Figure 3D). Results from PhyloNet with the Bayesian method also indicated that there was no reticulate evolutionary relationship among the redwoods (Supplemental Figure 6). These results suggested that ILS rather than hybridization led to topological inconsistencies among gene trees. Analysis of the syntenic gene set identified from syntenic blocks among the three redwood species also led to the same conclusions (Figures 3C, 3D, and S6).

### Genes related to growth characteristics of redwood species

**Figure 4:**
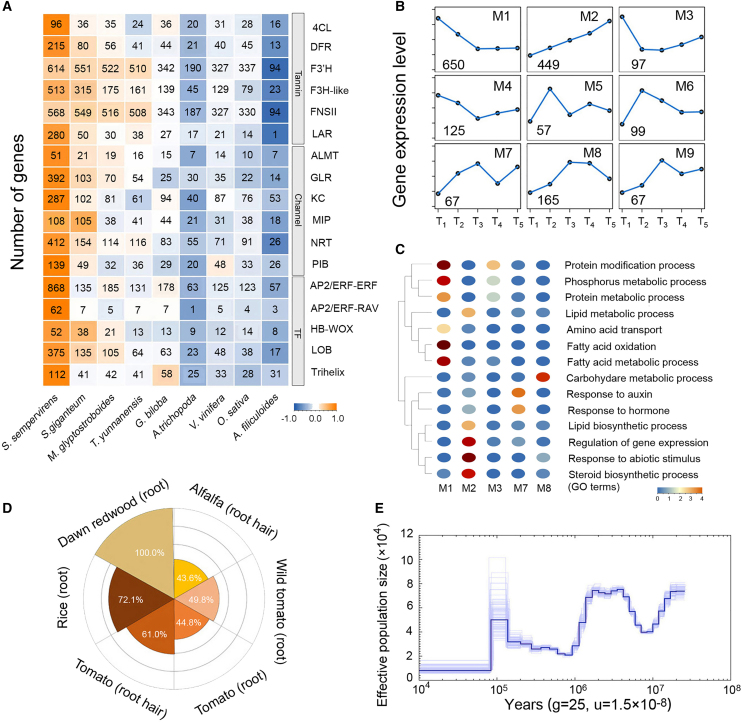
Gene families that control crucial biological traits and the conserved transcriptional response to flooding stress in *M. glyptostroboides.* **(A)** Expanded gene families in one or all redwood species that control crucial biological traits are compared with gene families in *T. yunnanensis*, *G. biloba*, *A. trichopoda*, *V. vinifera*, *O. sativa*, and *A. filiculoides*. The numbers of genes are normalized by gene family to a range of −1 to 1 for heatmap visualization. **(B)** The averaged expression patterns of genes in co-expression modules during flooding stress treatment. T1–T5 indicate 0, 3, 6, 9, and 12 h after submergence of *M. glyptostroboides* roots. M1–M9 indicate IDs of co-expression modules. **(C)** Gene Ontology enrichment of genes in co-expression modules (M1–M3 and M7–M8). **(D)** Numbers of orthologous genes between *M. glyptostroboides* and four other species regulated during flooding stress treatment at the transcriptional level. Data for species other than *M. glyptostroboides* were obtained from a previous study (Reynoso et al., 2019). **(E)** Estimated demographic history of *M. glyptostroboides*. The demographic modeling was scaled using an estimated synonymous substitution per site per year of 5.92216e−10 and a generation time of 25 years.

The genome sequences of *M. glyptostroboides* and its two relatives provide clues to the evolution of key growth characteristics of the three redwood species. Because *S. sempervirens is* a hexaploid, most gene families are expanded in *S. sempervirens* compared with *S. giganteum* and *M. glyptostroboides* (Figure 4A). Gene families involved in flavonoid/tannin biosynthesis pathways are expanded in *S. giganteum*, consistent with the high tannin concentration in its fire-resistant bark. The higher number of ion channel or transporter genes in *S. giganteum* and *S. sempervirens* may endow them with stronger water and nutrient uptake activity. Transcription factors (TFs) from the HB-WOX and LOB families are expanded in the three redwood species and may play roles in maintaining meristem activity. The expansion of AP2/ERF-ERF, AP2/ERF-RAV, and Trihelix families in *S. sempervirens* may be associated with adaptation to diverse stresses (Figure 4A).

### Conserved flooding response mechanism between gymnosperms and angiosperms

It is known that wetland crops have the adaptive potential to survive under submerged or flooded conditions. This adaptive potential can be tracked back to the eudicot–monocot split (Reynoso et al., 2019). *M. glyptostroboides* grows successfully in wetlands, indicating its physiological adaptation to flooding stress (Iwanaga et al., 2015; Yang et al., 2019). We performed RNA sequencing (RNA-seq) analysis to characterize the transcriptional response of *M. glyptostroboides* to flooding stress and check whether the signaling cascade is conserved between angiosperms and gymnosperms. About 1900 genes were differentially expressed between flooded and control roots of *M. glyptostroboides*. Co-expression analysis was performed, and the differentially expressed genes (DEGs) were grouped into 9 modules. Modules M1, M2, and M8 were the largest, containing 650, 449, and 165 genes, respectively (Figure 4B). Genes in M1 were downregulated in 6 h, whereas genes in M2 were induced gradually during the treatment process (Figure 4B). Genes involved in diverse metabolic processes (e.g., fatty acid metabolism, carbohydrate metabolism, and steroid biosynthesis) were over-represented in modules of the co-expression network (Figure 4C), which was consistent with various metabolic acclimation responses triggered during submergence (Phukan et al., 2016).

To compare the flooding response mechanisms of gymnosperms and angiosperms, transcriptome data from flooding stress experiments with several angiosperm species, including rice (a representative flood-resilient species), alfalfa (*Medicago truncatula*), domesticated tomato (*Solanum lycopersicum*), and wild tomato (*Solanum pennellii*, a dryland-adapted species) (Reynoso et al., 2019), were compared with those of flooded *M. glyptostroboides*. Among the 1877 DEGs in *M. glyptostroboides*, more than 72% were homologous to flooding-responsive genes in rice. The numbers of overlapping genes for alfalfa and the two tomato species were also significantly over-represented, but lower than those in rice (Figure 4D). These observations indicate that the flooding response at the transcriptome level is conserved in gymnosperm and angiosperm plants. During flooding stress, plants undergo many changes in architecture, energy metabolism, and endogenous phytohormone biosynthesis and signaling. The similarity in flooding stress perception and response in different species may result in similar reprogramming at the molecular level (Zhou et al., 2020).

### Historical fluctuations in effective population size of *M. glyptostroboides*

The fossil records indicated that *Metasequoia* species were distributed in wide regions of the northern hemisphere from the Cretaceous period (Stockey et al., 2001). Genomic data for *M. glyptostroboides* provide a resource for inference of population demographic history. The results showed a massive decline in effective population size (Ne) of *M. glyptostroboides* over the course of its evolution, with ∼90% extinction. Three major historical events that affected its population size were dated to about 10, 1, and 0.1 Mya (Figure 4E), corresponding to the middle Miocene climate transition (∼15–13.7 Mya), Xixiabangma (0.8–1.17 Mya), and Guxiang (0.13–0.3 Mya) in southeastern China (Mourik et al., 2011; Ren et al., 2020). The estimated current effective population size was approximately eight thousand, consistent with survey results from the 1970s (Ma, 2007).

## Discussion

Conifer genome sizes are typically larger than those of most animal and other plant species (Ahuja, 2005). About 63% of the *M. glyptostroboides* genome consists of repetitive sequences, including LTR retrotransposons. The accumulation of LTR TEs was continuous, with a large range of insertion times (8–150 Mya), older than that reported in *Pinus tabuliformis* (Niu et al., 2022). Phylogenetic analysis of the TEs indicated that amplification of *Copia*/*Gypsy* retrotransposons was clade specific (Figure 1E), consistent with results in *Taxus* (Xiong et al., 2021). Further characterization of how TEs affect the functions and regulation of protein-coding genes would aid in understanding the differentiation of redwood species. As proposed for *P. tabuliformis*, methylation of TEs may play roles in exon recognition during the process of gene expression (Niu et al., 2022).

The patterns of gene duplication events in *M. glyptostroboides* were analyzed, and a large number of dispersed, tandem, and TRD events were observed, which resulted in the expansion of gene families with diverse functions (Figure 1D and Supplemental Figure 3). The expansion of gene families associated with flavonoid/tannin biosynthesis, meristem maintenance, and ion/water transport were consistent with the growth traits of redwood species. *S. sempervirens* and *S. giganteum* are the tallest/largest trees on earth, and *M. glyptostroboides* can grow up to 40 m. Appropriate maintenance of meristem activity may contribute to the long life of redwood trees. Fire-resistant bark with high tannin concentrations can help mature trees to survive forest fires (Tributsch and Fiechter, 2008). Expansion of genes encoding ion channels may play a role in the fine control of guard cell movement for transpiration and water transport, which are essential for the physiological support of leaves at extreme heights (Kim et al., 2010).

The whole-genome annotations of *M. glyptostroboides*, together with those of *S. sempervirens* and *S. giganteum*, provide strong evidence for the phylogenetic relationships among redwood species and the origin of *S. sempervirens*. Duplicated genes in *S. sempervirens* clustered together in the phylogenetic trees, confirming that *S. sempervirens* is an autopolyploid, as analyzed using transcriptome data (Scott et al., 2016). A high ratio of incongruences between gene trees and the species tree was observed for single-copy genes. Previous transcriptome analysis of three redwood species revealed two minor topologies with similar CFs, indicating the contributions of ILS (Scott et al., 2016). Our phylogenetic analyses of single-copy genes using three independent software programs (QuIBL, MSCquartets, and PhyloNet) also indicated that ILS rather than hybridization led to the inconsistent phylogenies (Figure 3). The three extant redwood species exhibit both conserved and variant morphological and embryological characters (Ahuja and Neale, 2002). The lineage-specific mutations and morphologies could have been caused by the independent evolutionary trajectories of the three species. Some recent studies have also indicated that phylogenomic conflicts are closely associated with rapid morphological innovation (Parins-Fukuchi et al., 2021). In marsupials, more than 50% of the genome sequences are affected by ILS, and further comparative analysis and functional experiments indicated that ILS can affect complex morphological traits in extant species. Our results provide evidence for ILS during the evolution of redwoods and the formation of the ancient diploid *S. sempervirens* genome. Analyzing the relationship between ILS and morphological variations could help to determine whether morphological variations among redwood species are caused by random retention of ancestral polymorphisms in the ancestral populations.

Comparative genomic analysis of the three redwood species indicated that, in contrast to the highly collinear genomes of *M. glyptostroboides* and *S. giganteum*, the genome of *S. sempervirens* had undergone substantial chromosomal reorganization and/or genic fractionation and shared limited syntenic blocks with its relatives (Figure 2A). Substantial genome reorganization during diploidization may be a barrier to polyploidization in gymnosperms. Genomic resequencing using HiFi reads indicated that genomic rearrangements between *S. sempervirens* individuals were also large (Figure 2E). It has been proposed that diploidization after polyploidization is very slow in gymnosperms, which may explain the rarity of polyploidy in this clade (Scott et al., 2016). Slow diploidization results in continued multisomic inheritance and the postponement of evolutionary advantages. *S. sempervirens* was nonetheless able to avoid extinction, perhaps because of some key traits such as clonal reproduction, self-compatibility, and extreme longevity (Scott et al., 2016). In addition to this hypothesis, our analysis suggests that dramatic genome reorganization during diploidization may result in the accumulation of deleterious mutations, and only a limited number of gymnosperm species can survive the genome shock. As reported previously, chromosomes in *S. sempervirens* meiotic cells are mostly in a bivalent state, but a few are in a multivalent state (Ahuja and Neale, 2002). It has been proposed that *S. sempervirens* may be a complex species with different types of hexaploids and distinct genotypes (Ahuja and Neale, 2002). The significant structural variation between *S. sempervirens* individuals could be caused by the independent process of diploidization during evolution and selection. There is a paradox about the chromosome structures of polyploids (Jiang et al., 2021): rapid genomic reshuffling has been observed in many species like *Brassica napus* (Xiong et al., 2011) and *Tragopogon miscellus* (Chester et al., 2012), but other species like *Arabidopsis suecica* (Burns et al., 2021; Jiang et al., 2021) and cotton (*Gossypium*) (Chen et al., 2020) exhibit genomic stability. Genomic and epigenomic changes in sub-genomes may improve chromosome stability (Jiang et al., 2021). Currently, our HiFi assembly of *S. sempervirens* is at the contig level, and the previously published reference genome is at the scaffold level (Neale et al., 2021). Chromosome-scale genome sequences of more *S. sempervirens* individuals will provide further insights into the genomic rearrangement of *S. sempervirens* after polyploidization.

Our comparative analysis provided evidence for ILS during redwood evolution. Further analysis of genome sequences of redwood species at the population level, especially the features of centromere regions, could provide more details about the evolutionary process. On the basis of the genomic resources, multi-omics data could be collected to further understand the evolution and adaptation of redwood species. The genome sequence of *M. glyptostroboides* can also facilitate conservation of this threatened species through different approaches. The genetic diversity of the extant population can be evaluated using the reference to screen representative individuals to maximize the gene pool. Major loci that control fertility can be identified to guide breeding efforts to improve the production and germination ability of hybrid seeds.

## Methods

### DNA extraction, library construction, and sequencing

Fresh leaves of dawn redwood (*M. glyptostroboides*) were collected from Wuhan, Hubei Province, China. The leaves were thoroughly washed with distilled water, and high-quality genomic DNA was extracted using the CTAB method (Porebski et al., 1997). All sequencing experiments were performed at Wuhan Benagen Tech Solutions Company Ltd. (Wuhan, China) unless otherwise specified.

For short-read sequencing, paired-end libraries were prepared using the Nextera DNA Flex Library Prep Kit (Illumina, San Diego, CA) with an insert size of 300 bp and sequenced on the Illumina NovaSeq platform (Illumina). The raw reads were filtered using SOAPnuke (version 2.1.4) (https://github.com/BGI-flexlab/SOAPnuke) to remove low-quality reads. Reads containing adaptors or unknown nucleotides (Ns) or reads with >20% low-quality bases were removed. The filtered clean data were used for subsequent data processing and bioinformatic analyses.

For ONT genomic sequencing, libraries were constructed using the SQK-LSK109 ligation kit and sequenced on a PromethION sequencer (ONT, Oxford, UK) with 48-h runs by loading onto primed R9.4 Spot-On Flow Cells. Base calling analysis of the raw nanopore sequencing data was performed using ONT Guppy software (version 0.3.0).

For Hi-C library construction and sequencing, *M. glyptostroboides* leaves were collected and cross-linked using formaldehyde. The genomic DNA was extracted and treated with MboI enzyme. The cohesive ends were filled and used for blunt-end ligation. After ligation, the cross-linking was reversed, and the DNA was purified and sheared to a length of ∼400 bp. Point ligation junctions were pulled down and used for sequencing library construction as described previously (Ramani et al., 2020). The final library was sequenced using 150-bp paired-end mode on the Illumina NovaSeq sequencing platform.

For ONT cDNA sequencing, total RNA was isolated from leaf, stem, bud, cambium, and root tissues using the Direct-zol RNA kit (Zymo Research, Irvine, CA). The quantity and quality of RNA were assessed using a Nanodrop 2000 ultraviolet spectrophotometer (Thermo Fisher Scientific) and an Agilent Bioanalyzer 4200 system (Agilent Technologies, Santa Clara, CA). RNA samples of equal quantity were mixed for PCR-cDNA library construction using a Ligation Sequencing Kit (SQK-LSK109) and sequenced on the PromethION sequencer (ONT). Base calling analysis was performed using ONT Guppy software (version 0.3.0).

For PacBio HiFi genome sequencing of coast redwood (*S. sempervirens*), a leaf sample was collected from Nanjing, Jiangsu Province, China. High-quality genomic DNA was extracted using the CTAB method. The quantity and quality of DNA were assessed as described above. The high-molecular-weight genomic DNA was sheared using a Megaruptor (Diagenode, Denville, NJ) to obtain a size distribution between 15 and 20 kb. The HiFi sequencing library was prepared using the SMRTbell Express Template Prep Kit 2.0 and sequenced using the PacBio Sequel II system according to the manufacturer’s instructions (Pacific Biosciences, CA, USA). SMRT Link version 11.0 was used to generate HiFi reads from the subread file.

### Genome assembly

The genome size of *M. glyptostroboides* was estimated by flow cytometry with *Liriodendron chinense* (1n = 1.8 Gb) as the control and by K-mer analysis of the Illumina short reads with Jellyfish (version 2.3.0) (Marçais and Kingsford, 2011) and GenomeScope (version 2.0) (Ranallo-Benavidez et al., 2020) (Supplemental Figure 1). Genome assembly was performed with SMARTdenovo (https://github.com/ruanjue/smartdenovo) using the Nanopore sequencing data. Two rounds of error correction were performed using the Nanopore and Illumina NovaSeq sequencing data with Racon (version 1.4.11, https://github.com/isovic/racon) and Pilon (version 1.23) (Walker et al., 2014), respectively. Heterozygous sequences were removed using the Purge_haplotigs pipeline (version 1.0.4) (Roach et al., 2018). The completeness of the genome assembly was evaluated using BUSCO (version 4.1.2) (Simao et al., 2015), Merqury software (Rhie et al., 2020), and the mapping rate of Illumina short reads aligned to the assembled genome. We used ALLHIC (version 0.9.12) and 3D-DNA (version 180419) for pseudochromosome-level scaffolding and stitching, respectively, then imported the files into Juicebox (version 1.11.08) (Robinson et al., 2018) to prepare plots. The *S. sempervirens* genome was assembled using Hifiasm with default settings (Cheng et al., 2021).

### Genome annotation of *M. glyptostroboides*

Repeat sequences were annotated with RepeatModeler (version 1.0.4, https://github.com/rmhubley/RepeatModeler) and RepeatMasker (version 4.0.5, http://www.repeatmasker.org/) (Tarailo-Graovac and Chen, 2009). GenomeTools (version 1.5.9) (Gremme et al., 2013) was used to detect full-length LTR retrotransposons. LTRs from the *Copia* and *Gypsy* super-families were identified on the basis of the functional domains and their orders. Phylogenetic trees were generated in FastTree (version 2.1.9) (Price et al., 2010) after aligning the amino acid sequences in MUSCLE (version 3.8.3.1) (Edgar, 2004). The insertion times (t) of intact LTRs were estimated as t = K/2r, where the rate of nucleotide substitution (r) used for redwood species was 5.92216e−10 (De La Torre et al., 2017).

Protein-coding genes were annotated by a combination of transcript mapping, *ab initio* gene prediction, and homologous gene alignment. In brief, the ONT cDNA reads were mapped to the reference genome with Minimap2 (version 2.17) (Li, 2018), and the transcripts were assembled using StringTie2 (version 2.1.5) (Pertea et al., 2015). The ORFs were predicted using TransDecoder (version 5.1.0), and *ab initio* gene prediction was performed using Augustus (version 3.3.2) (Nachtweide and Stanke, 2019), GENSCAN (version 1.0), and GlimmerHMM (version 3.0.4). We then performed homologous gene alignment by aligning proteins from related species (*T. yunnanensis*, *Pseudotsuga menziesii*, and *S. giganteum*) using Exonerate (version 2.4.0). MAKER (version 2.31.10) was used to integrate the results of gene prediction, and genes with incomplete structures or short CDSs (<150 bp) were removed. The quality of the gene predictions was evaluated using BUSCO (version 4.1.2) (Simao et al., 2015) with the eukaryotic and embryophyte database. The genomic features of protein-coding genes and repetitive elements were visualized as a Circos plot using the circlize package (version 0.4.15).

### Gene function annotation of *M. glyptostroboides*

Multiple databases were used to annotate the functions of protein-coding genes. Blastp (E-value cut-off 1e−05) was performed against the NCBI NR and UniProt databases. Protein motifs and domains were searched using InterProScan (version 5.33) (Jones et al., 2014) and HMMER (version 3.1). Gene Ontology (GO) terms were obtained from either InterPro (https://github.com/ebi-pf-team/interproscan) or UniProt entries, and Kyoto Encyclopedia of Genes and Genomes (KEGG) pathway annotation was performed in KOBAS (version 3.0, https://github.com/xmao/kobas).

### Phylogenetic analysis and gene family expansion and contraction in nine species

Single-copy OGs in nine species were identified using OrthoFinder (version 2.5.4) (Emms and Kelly, 2019), and 195 groups were subjected to multiple sequence alignment with mafft (version 6.864) (Nakamura et al., 2018). RAxML (version 8.2.10) (Stamatakis, 2014) was used to reconstruct phylogenetic trees for each gene family. Maximum likelihood (ML) trees were inferred in ASTRAL (Mirarab et al., 2014) with 100 bootstraps using the supertree method.

Divergence times were estimated from the phylogenetic trees using r8s software (version 1.71). Previously estimated divergence times for different model and non-model plants were used to calibrate the divergence times: *Azolla filiculoides* and *Vitis vinifera* (392–422 Mya), *Ginkgo biloba* and *T. yunnanensis* (271–310 Mya), and *Amborella trichopoda* and *Oryza sativa* (173–199 Mya). The gene family clustering results were used to analyze gene family contraction and expansion with CAFÉ (version 2.1) (Han et al., 2013). Gene numbers in key gene families were confirmed by searching for the conserved protein domains.

### Phylogenetic analysis and ILS and hybridization test of redwood genomes

Orthologous groups (OGs) were identified in the three redwood species and *T. yunnanensis* using OrthoFinder (version 2.5.4). The OGs were visualized with a Venn diagram and used for screening of OGs with different patterns. Genes from OGs with single-copy genes in *M. glyptostroboides*, *S. giganteum*, and *T. yunnanensis* were extracted for phylogenetic analysis. Multiple sequence alignments of the CDSs were obtained using mafft (version 6.864), and ML trees were constructed with RAxML (version 8.2.10) using the GTRGAMMA model with 1000 bootstrap replicates. OGs with high-confidence bootstrap support values (≥50) were selected for further analysis. Alignment results for OGs with one, two, and three gene copies in *S. sempervirens* are provided in Supplemental Data 1–3, respectively. The topologies of the phylogenetic trees were classified into nine major types.

Two sets of genes were used for the ILS and hybridization test: (1) single-copy genes in four species from OGs identified using OrthoFinder and (2) syntenic genes in syntenic blocks of the three redwood species. The syntenic blocks were identified using WGDI (Sun et al., 2022) with the command “wgdi -icl” using GFF files of genome annotations and blast results from a pair of genomes as inputs. The alignment results for genes from syntenic blocks are given in Supplemental Data 4.

Three software tools were used in our analysis: MSCquartets (Analyzing Gene Tree Quartets under the MSC) (Rhodes et al., 2020), QuIBL (Quantifying Introgression via Branch Lengths) (Edelman et al., 2019), and PhyloNet (evaluating reticulate evolutionary relationships using a Bayesian method) (Wen et al., 2018). The T3 model was used in MSCquartets, and the sampleFrequency parameter was set to 10 000 for PhyloNet. Default settings were used for QuIBL.

### Collinearity and whole-gene duplication analysis

Genomic collinearity and gene duplication analyses were performed using MCScan (Python version, https://github.com/tanghaibao/jcvi/wiki/MCScan-(Python-version)) (Wang et al., 2012). The amino acid sequences of different species were self-aligned with Blastp. The Ks values were calculated using the PAML package (Yang, 2007) and represented as median values, and the distribution of corrected Ks values was plotted. DupGen_finder was used to classify gene duplication events on the basis of their chromosomal positions (Qiao et al., 2019).

### Flooding stress treatment and transcriptome analysis

One-year-old *M. glyptostroboides* seedlings (height = 60 cm) were subjected to flooding treatment. Plants germinated from seeds were planted in soil with organic culture substrate in plastic containers (50 × 85 cm). The plants were watered well before the treatment and then maintained for 24 h. After 24 h, the plants were flooded to 10 cm above the soil level. The taproots were collected in triplicate samples at 0, 3, 6, 9, and 12 h after flooding treatment. The roots were immediately frozen in liquid nitrogen, then stored at −80°C until further use.

Total RNA was extracted using the Direct-zol RNA kit (Zymo Research) and used for Illumina RNA-seq. Sequencing was performed at ANNOROAD Gene Technology, Beijing, China. Sequence reads were quality checked using fastqc version 0.11.9 and mapped onto the reference genome using STAR version 2.7.9a. RNA-seq reads of matching genes were calculated using RSEM (Li and Dewey, 2011). The ANOVA-like method of the edgeR package was used to identify DEGs between time points. Co-expression network analysis was performed using WGCNA (Langfelder and Horvath, 2008), and the gene sets in each module were subjected to GO enrichment analysis using the clusterProfiler R package (version 4.6) (Yu et al., 2012). Gene expression data from flooding-stressed angiosperm plants were downloaded from Reynoso et al. (2019). Homologous gene pairs between *M. glyptostroboides* and the compared species were identified using a blast search.

### Estimation of demographic history

Paired-end *M. glyptostroboides* sequence reads were mapped to the assembled genome using the BWA-MEM algorithm. Aligned reads with high mapping scores (>20) were screened and sorted with Picard tools (version 1.95; http://broadinstitute.github.io/picard/). The evolutionary effective population size of *M. glyptostroboides* was inferred using PSMC (https://github.com/lh3/psmc). The results of the demographic modeling were scaled using an estimated synonymous substitution per site per year of 5.92216e−10 (De La Torre et al., 2017) and a generation time of 25 years.

## Data availability

Sequences and annotations of the *M. glyptostroboides* genome have been deposited in the China National Center for Bioinformation (CNCB, https://ngdc.cncb.ac.cn) under accession number PRJCA016596. All raw data from *M. glyptostroboides* and *S. sempervirens* have also been deposited. In detail, raw reads from the genomic library sequenced using the ONT PromethION platform and raw reads from Hi-C and genomic resequencing libraries sequenced using the Illumina NovaSeq 6000 platform have been deposited in the China National GeneBank DataBase (CNGBdb) (https://db.cngb.org/cnsa), CNGB Sequence Archive (CNSA), under accession number CNP0003114. Raw reads from the ONT cDNA library and RNA-seq reads from the Illumina NovaSeq 6000 platform have been deposited in the CNGBdb under accession number CNP0004335. The genome assembly of *S. sempervirens* and the unassembled raw reads from the PacBio HiFi genomic library have also been deposited under accession number CNP0004335 (CNGBdb). Descriptions of samples and libraries are provided in Supplemental Table 14.

## Funding

This research was supported by the 10.13039/501100012166National Key Research and Development Program of China (2017YFD0600701).

## Author contributions

Conceptualization, F.C., F.F., and L.X.; Methodology, F.F., C.S., C.W., L.Y., and L.X.; Investigation, L.X., F.F., C.S., C.W., L.Y., Y.G., X.Y., Z.S., Y.F., B.L., M.S., Y.Z., L.C., Y.N., and J.C.; Writing – Original Draft, F.F. and L.X.; Writing – Review & Editing, F.F., L.X. and F.C.; Funding Acquisition, F.C.; Resources, F.F., C.S., X.L., and G.W.; Supervision, F.C., L.X., S.C., and T.Y.

## Supplemental information

Document S2. Supplemental Data 1–4

Document S3. Article plus supplemental information
