# Supplementary material for: The Metasequoia genome and evolutionary relationships among redwoods
Source: Plant Commun. 2023 Jun 28;4(6):100643. doi: 10.1016/j.xplc.2023.100643 (PMC10775903; doi:10.1016/j.xplc.2023.100643)
Supplement: Document S1. Supplemental Figures 1–6 and Supplemental Tables 1–14 [file mmc1.pdf]

Plant Communications, Volume *4*

Supplemental information

The *Metasequoia* genome and evolutionary relationships among redwoods

Fangfang Fu, Chi Song, Chengjin Wen, Lulu Yang, Ying Guo, Xiaoming Yang, Ziqiang Shu, Xiaodong Li, Yangfan Feng, Bingshuang Liu, Mingsheng Sun, Yinxiao Zhong, Li Chen, Yan Niu, Jie Chen, Guibin Wang, Tongming Yin, Shilin Chen, Liangjiao Xue, and Fuliang Cao

The *Metasequoia* genome and evolutionary relationship among redwoods

Fangfang Fu [1†], Chi Song[2,3†], Chengjin Wen[1†], Lulu Yang[3†], Ying Guo[1], Xiaoming Yang[1], Ziqiang Shu[3], Xiaodong Li[4], Yangfan Feng[1], Bingshuang Liu[1], Mingsheng Sun[1], Yinxiao Zhong[1], Li Chen[1], Yan Niu[3], Jie Chen[3], Guibin Wang[1], Tongming Yin[1]*, Shilin Chen[5]*, Liangjiao Xue [1]*, Fuliang Cao[1]*

[1]*State Key Laboratory of Tree Genetics and Breeding, Co-Innovation Center for Sustainable Forestry in Southern China, Nanjing Forestry University, Nanjing 210037, China;*
[2]*Institute of Herbgenomics, Chengdu University of Traditional Chinese Medicine, Chengdu 611137, China;*
[3]*Wuhan Benagen Technology Company Limited, Wuhan 430000, China;*
[4]*Wuhan Botanical Garden, Chinese Academy of Sciences, Wuhan 430074, China;*
[5]*China Academy of Chinese Medical Sciences, Institute of Chinese Materia Medica, Beijing 100070, China*

[†]Contributed equally to this work
*Corresponding authors (Fuliang Cao, email: fuliangcaonjfu@163.com; Liangjiao Xue, email: lxue@njfu.edu.cn; Shilin Chen, email: slchen@icmm.ac.cn; Tongming Yin, email: tmyin@njfu.edu.cn)

Supplemental Information

6 supplementary figures
14 supplementary tables

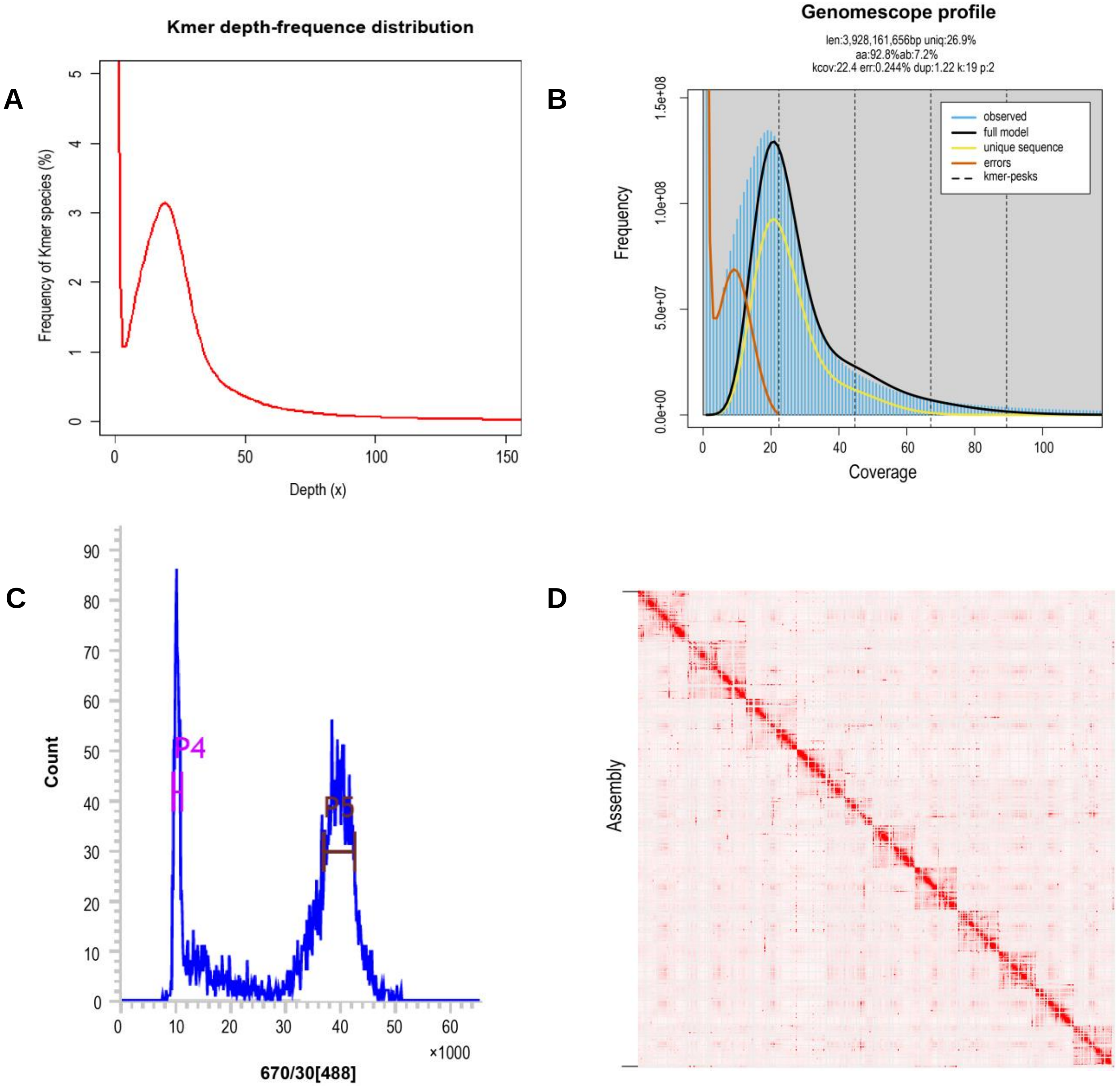

Supplementary Figure S1 The genome estimation of *Metasequoia glyptostroboides*.
(A) The distribution of k-mer count.
(B) GenomeScope analysis based on the statistics of 19-mers.
(C) Genome size estimation by flow cytometer. *Liriodendron chinense* was used as standard (n=1.8G). P4 and P5 were the peaks of *L. chinense* and *M. glyptostroboides*, respectively.
(D) The Hi-C interactive heat map of *M. glyptostroboides*.

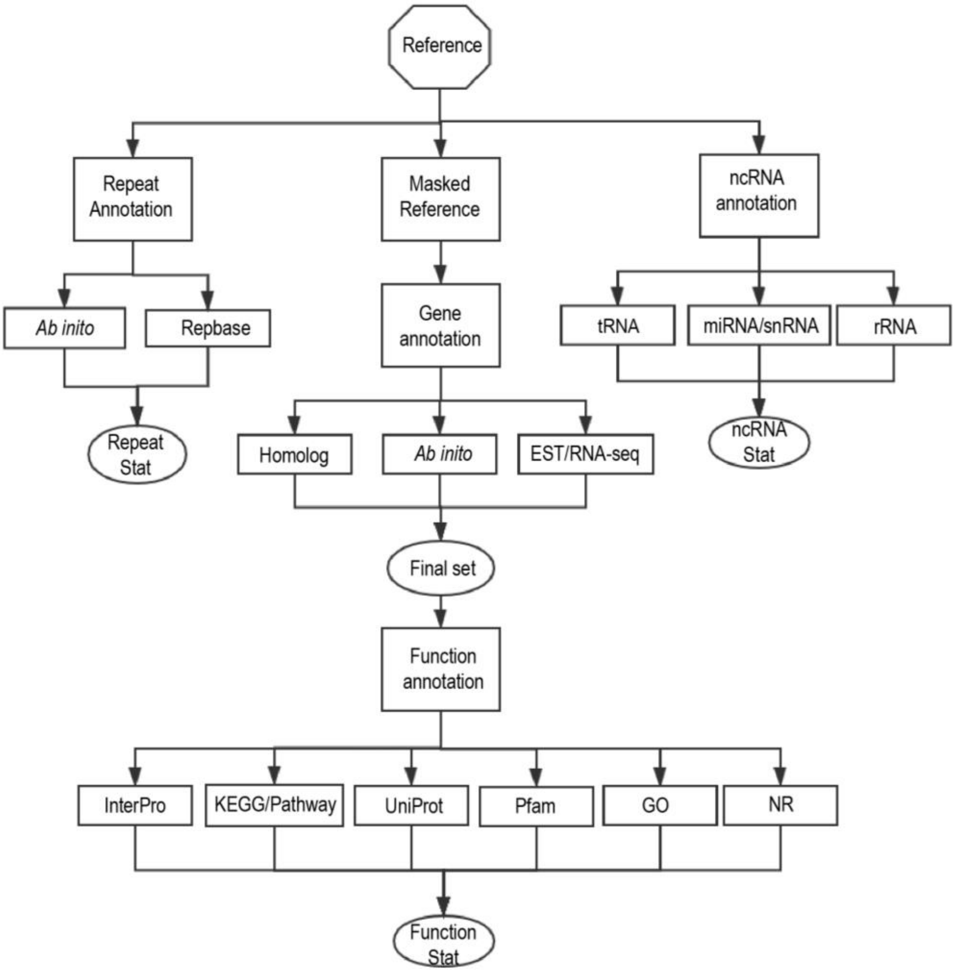

Supplementary Figure S2 The flowchart of genome assembly and annotation for *M. glyptostroboides.*

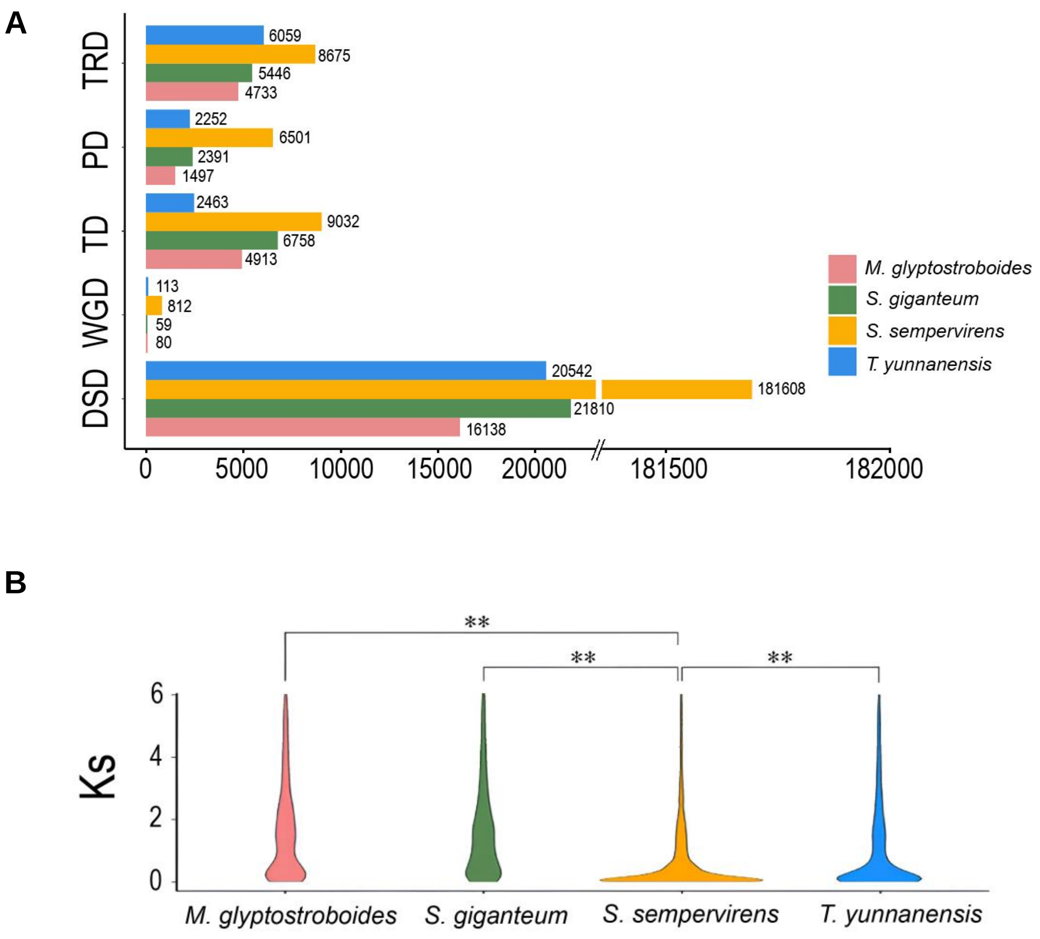

Supplementary Figure S3 Gene duplication events in redwood species.
(A) Categories and numbers of different gene duplication events in redwoods and the outgroup *Taxus yunnanensis*. TRD, transposed duplication; PD, proximal duplication; TD, tandem duplication; WGD, whole-genome duplication； DSD, dispersed duplicate.
(B) Violin plots showing the synonymous mutation rates (Ks) of genes originated from transposed duplication (TRD) in redwood species and *T. yunnanensis*. Asterisks denote the significant difference ($P < 0.001$) with Wilcoxon rank-sum test.

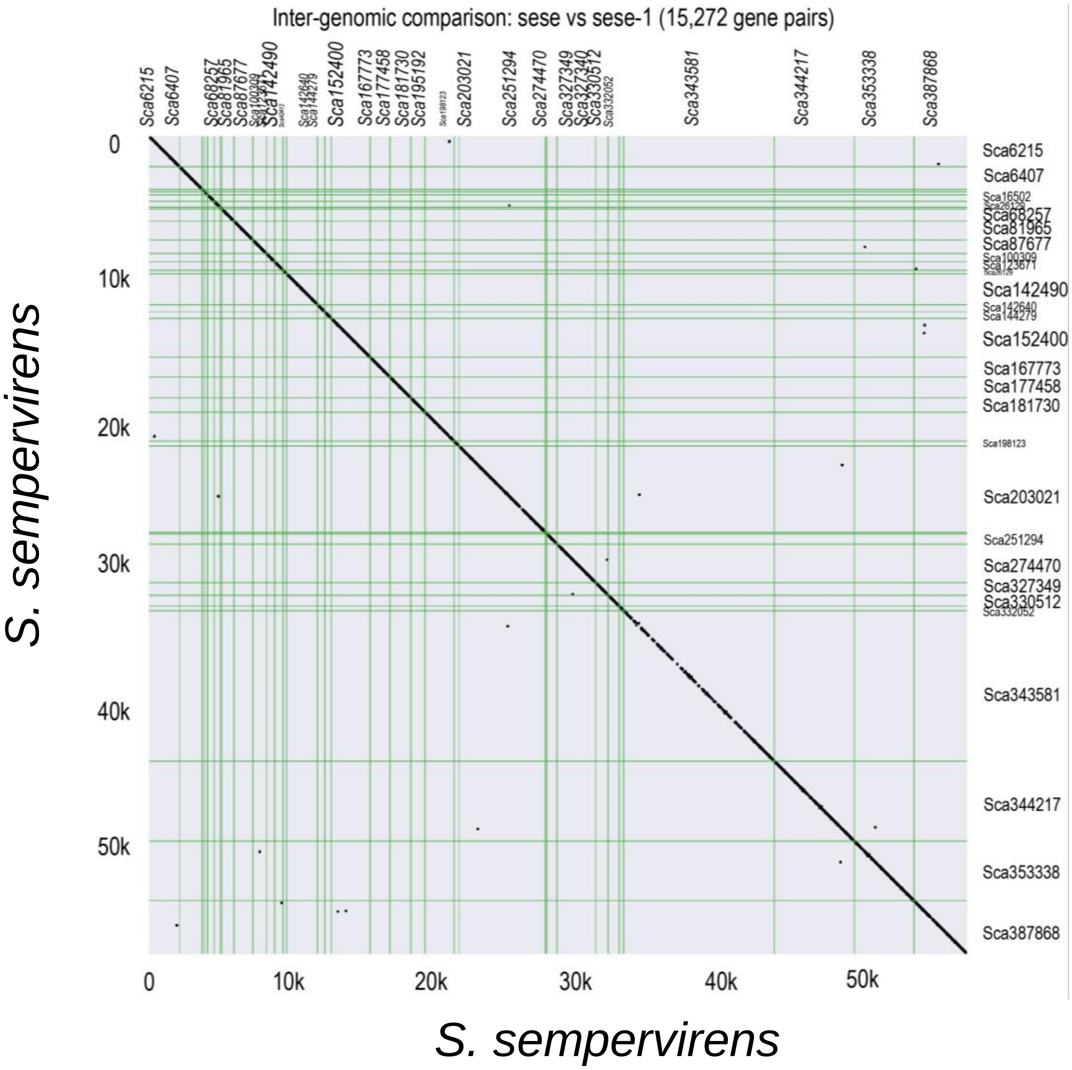

Supplementary Figure S4 Syntenic dot plot of *Sequoia sempervirens* genome.

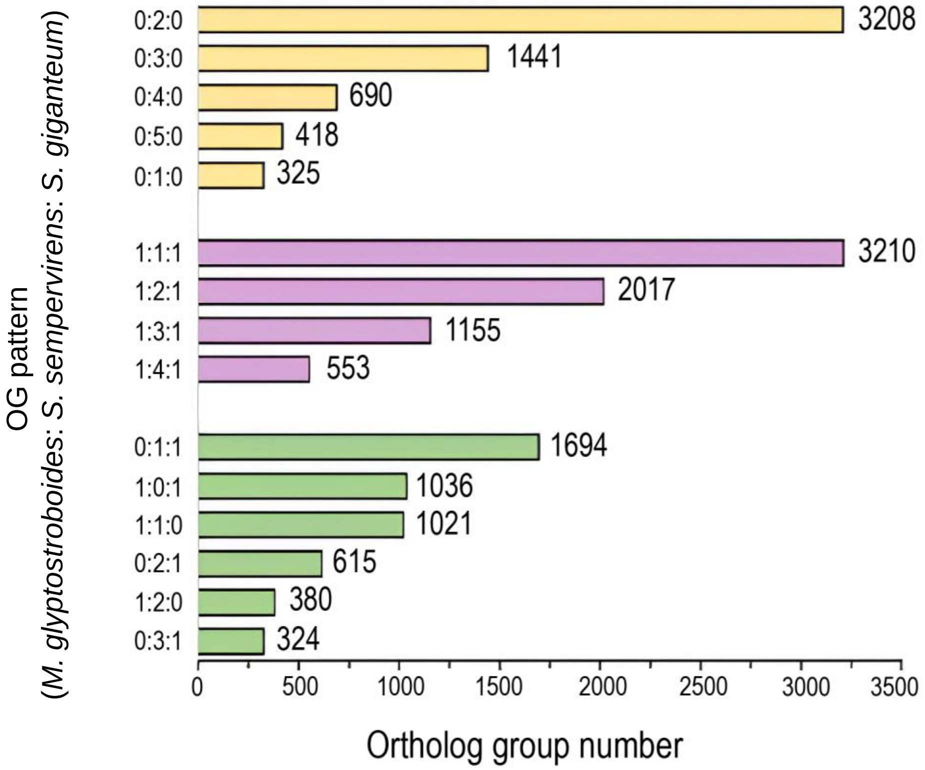

Supplementary Figure S5 Numbers of ortholog groups with different patterns of copy number as in *M. glyptostroboides: S. sempervirens: S. giganteum*.

Single-copy set

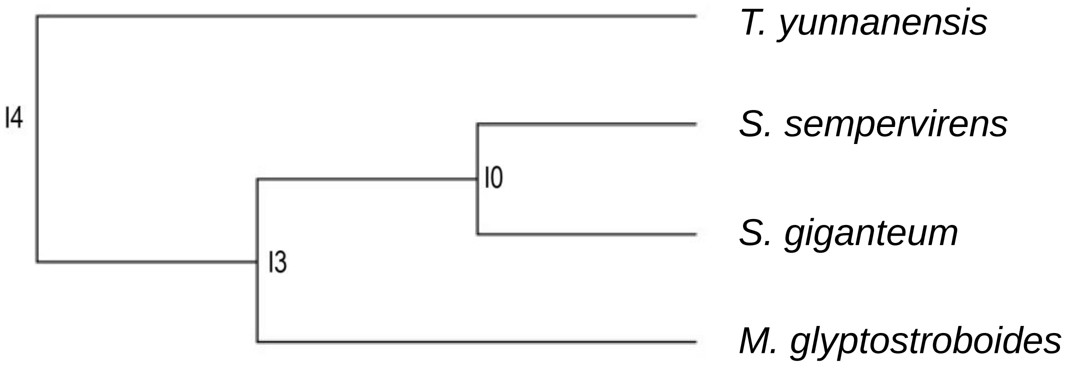

Overall MAP = -1793.354493712107

Synteny set

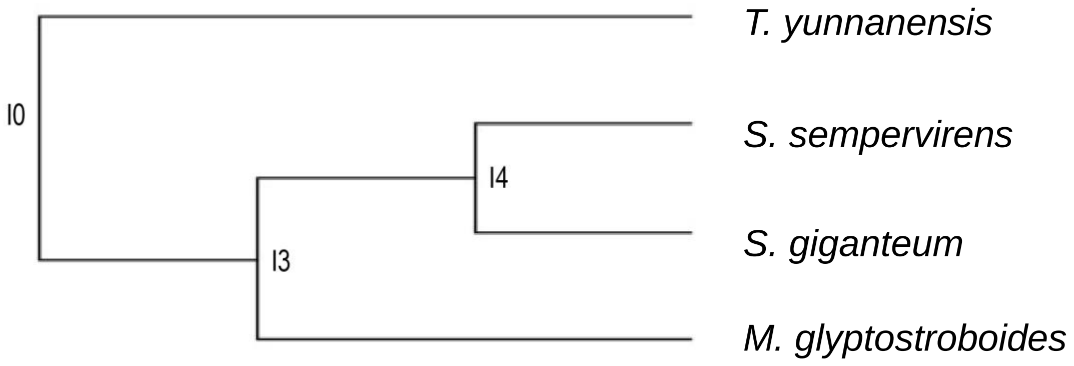

Overall MAP = -337.7857678737349

Supplementary Figure S6 Reconstructing of phylogenetic networks of redwood species using PhyloNet. Single-copy and Synteny gene sets were analyzed independently.

Table S1. Clean data of Oxford Nanopore long-reads.

Rank	Flag	Total Base (bp)	Total Reads	Max_Len (bp)	Avg_Len (bp)	N50 (bp)	N90 (bp)	Mean Q
	all	529,761,024,708	22,724,907	1,185,616	23,312	35,628	15,009	8.48
>0K	pass	429,941,519,134	16,633,745	305,492	25,848	36,339	15,587	9.76
	fail	99,819,505,574	6,091,162	1,185,616	16,388	32,613	12,701	4.98
	all	523,040,610,927	18,377,312	1,185,616	28,461	35,942	15,669	8.89
>5K	pass	426,120,281,201	14,643,698	305,492	29,099	36,561	16,055	9.81
	fail	96,920,329,726	3,733,614	1,185,616	25,959	33,310	14,152	5.25
	all	512,161,880,475	16,847,324	1,185,616	30,400	36,456	16,719	8.94
>10K	pass	418,597,430,939	13,580,992	305,492	30,822	36,997	16,964	9.83
	fail	93,564,449,536	3,266,332	1,185,616	28,645	34,128	15,768	5.28
	all	129,015,254,841	2,058,086	1,185,616	62,687	60,910	51,762	9.17
>50K	pass	109,306,794,065	1,740,960	305,492	62,785	61,039	51,789	9.85
	fail	19,708,460,776	317,126	1,185,616	62,147	60,228	51,627	5.44

Table S2. Data statistics of Illumina reads.

Type	Total Base (bp)	Total Reads	GC(%)	Q20(%)	Q30(%)
Raw	256,036,361,100	1,706,909,074	34.85	98.12	94.41
Clean	253,554,657,329	1,693,803,066	34.7	98.38	94.75

Table S3. Statistics of Hi-C sequencing data.

Item	Value
Clean Reads	2,304,824,114
Clean Paired-end Pairs	1,152,412,057
Unique Mapped Paired-end Pairs	324,185,700
Unique Mapped Ratio	28.1%
Dangling End Paired-end Pairs	77,296
Self Circle Paired-end Pairs	490,745
Valid Paired-end Pairs	315,868,371
Valid Rate	97.43%

Table S4. Hi-C aided chromosome-scale scaffolding of the assembly for *M. glyptostroboides*. Chromosomes are numbered according to the collinearity with *S. giganteum*.

Chromosome	Length (bp)	Contig number
Chr1	818,874,304	141
Chr2	883,923,269	101
Chr3	973,530,065	130
Chr4	722,600,678	86
Chr5	860,062,275	123
Chr6	715,836,781	105
Chr7	702,647,723	83
Chr8	628,347,089	86
Chr9	646,074,236	97
Chr10	622,644,211	64
Chr11	452,563,994	43
Unanchored	47,979,437	63

Table S5. BUSCO statistics of genome assembly assessing.

Item	Number	Percent (%)
Complete BUSCOs (C)	1,321	81.9
Complete and single-copy BUSCOs (S)	1,244	77.1
Complete and duplicated BUSCOs (D)	77	4.8
Fragmented BUSCOs (F)	93	5.8
Missing BUSCOs (M)	200	12.3
Total BUSCO groups searched	1,614	100

Table S6. Comparison of BUSCO statistics between some selected species.

Species	Genome size (Gb)	Gene number	Genome assembly BUSCO (1614)	Gene sets BUSCO (1614)
Gnetum montanum	4.07	27,491	74.40%	83.80%
Ginkgo biloba	9.87	27,832	45.30%	63.70%
Pseudotsuga menziesii	15.70	51,419	52.10%	68.50%
Picea abies	19.60	70,736	32.00%	28.10%
Pinus lambertiana	31.00	38,518	36.90%	73.30%
Pinus taeda	20.10	51,751	39.70%	41.70%
Taxus yunnanensis	10.73	30,766	52.50%	52.00%
Sequoiadendron giganteum	8.13	37,936	38.40%	50.00%
Metasequoia glyptostroboides	**8.07**	**32,184**	**65.30%**	**81.90%**

Table S7. Gene structure annotation using multiple methods.

Method	Software	Species	Gene number	Average Length (bp)				Average exon per gene
				Gene	CDS	Exon	Intron	
Ab initio	GlimmmerHMM		419,161	18,988	495	108	5,171	4.58
Ab initio	AUGUSTUS		171,798	5,888	772	229	2,158	3.37
Homology-based	Exonerate	*Pseudotsuga menziesii*	440,773	18,206	579	330	23,338	1.76
Homology-based	Exonerate	*Sequoiadendron giganteum*	1,867,347	13,895	536	337	22,592	1.59
Homology-based	Exonerate	*Taxus yunnanensis*	634,183	19,836	557	328	27,541	1.70
RNAseq	TransDecoder		17,236	34,474	1,347	466	6,544	5.85
Integration	Maker		105,089	14,993	767	189	3,906	4.62
Final set	Anno-self		**32,184**	21,925	1,205	360	5,207	4.87

Table S8. Functional annotation of protein-coding genes.

Item	Count	Percentage
Protein models	32,184	-
Annotation	30,149	93.68%
Uniprot	27,096	84.19%
Pfam	25,425	79.00%
GO	23,669	73.54%
KEGG	11,930	37.07%
Pathway	7,511	23.34%
Interproscan	29,667	92.18%
NR	24,668	76.65%

Table S9. Summary of gene pairs in synteny blocks between *M. glyptostroboides* and *S. giganteum*, *S. sempervirens*, *T. yunnanensis* and *G. biloba*, respectively.

Type	Length (bp)	Number	Average Length (bp)
rRNA	86,689	302	287.05
tRNA	477,517	6,566	72.7257
ncRNA	1,302,877	10,729	121.435

Table S10. The proportion of repetitive sequences in the genome of different species.

Type	*Metasequoia glyptostroboides*	*Sequoiadendron giganteum*	*Taxus yunnanensis*	*Ginkgo biloba*	*Gnetum montanum*	*Pseudotsuga menziesii*	*Pinus taeda*	*Oryza sativa*	*Vitis vinifera*	*Azolla filiculoides*	*Amborella trichopoda*
DNA	7.3	5.65	7.15	2.97	1.08	4.95	3.5	14.97	5.33	3.16	16.31
LINE	2.65	3.53	5.39	3.89	17.26	3.98	4.2	1.22	16.77	8.13	9.28
SINE	0.06	0.07	0.02	0	0.59	0.02	0	0.12	0.03	0.19	0.12
LTR	28.27	21.75	24.45	41.45	14.75	36.5	32.4	6.88	14.82	9.42	14.31
Satellite	0.15	0.1	0.03	0.1	0.04	0.13	0.2	0.02	0.03	0.17	0.07
Simple_repeat	0	0	0	0	0	0.02	0	0	0	0	0
Other	0	0	0	0	0	0	0	0	0	0	0
Unknown	25.53	26.52	26.61	28.95	24.93	25.31	22.49	0.4	3	7.62	3.8
Total	62.96	57.1	62.52	74.55	57.88	69.18	60.96	24.41	40.46	29.7	44.66

Table S11. Summary of gene pairs in synteny blocks between *M. glyptostroboides* and *S. giganteum*, *S. sempervirens*, *T. yunnanensis* and *G. biloba*, respectively.

Item	*M.gly - S.gig*	*M.gly - S.sem*	*M.gly - T.yun*	*M.gly - G.bil*
Gene pairs	14,132	11,031	9,008	7,857
Block No.	94	828	391	534
Mean	150.3	13.3	23	14.7
Medium	65	10	10	11
Max	970	102	264	94

Table S12. Different patterns of gene copy number in *S. sempervirens* of Ortholog groups (OGs) with single gene in *M. glyptostroboides* and *S. giganteum*, and the outgroup *T. yunnanensis.*

Pattern	Gene Copy Number				Gene Number	Gene Ratio (%)
	M.gly	*S.sem*	*S.gig*	*T.yun*		
1:1:1:1	1	1	1	1	2,258	36.84
1:2:1:1	1	2	1	1	1,596	26.04
1:3:1:1	1	3	1	1	958	15.63
1:0:1:1	1	0	1	1	536	8.74
1:4:1:1	1	4	1	1	449	7.32
1:5:1:1	1	5	1	1	187	3.05
1:6:1:1	1	6	1	1	67	1.09
1:7:1:1	1	7	1	1	41	0.67
1:8:1:1	1	8	1	1	12	0.20
1:9:1:1	1	9	1	1	7	0.11
1:11:1:1	1	11	1	1	6	0.10
1:10:1:1	1	10	1	1	4	0.07
1:14:1:1	1	14	1	1	2	0.03
1:12:1:1	1	12	1	1	1	0.02
1:13:1:1	1	13	1	1	1	0.02
1:15:1:1	1	15	1	1	1	0.02
1:16:1:1	1	16	1	1	1	0.02
1:22:1:1	1	22	1	1	1	0.02
1:25:1:1	1	25	1	1	1	0.02
1:75:1:1	1	75	1	1	1	0.02
Total					6,130	100.00

Table S13. Statistics of QuIBL analysis.

Gene Set	Triplet	Out group	C1	C2	mixprop1	mixprop2	lambda2 Dist	lambda1 Dist	BIC2Dist	BIC1Dist	Count	totalIntro Prop	Pair
Single-Copy Set	2_1_3	2	0	0.1663	0.7248	0.2752	0.0094	0.0096	-3438.5066	-3448.7310	474	0.0642	3_1
	2_1_3	1	0	0.2911	0.7513	0.2487	0.0117	0.0120	-7173.2471	-7194.9010	1052	0.1287	2_3
	2_1_3	3	0	0.0440	0.8327	0.1673	0.0187	0.0188	-2995.6960	-3010.8403	507	0.0417	2_1
Synteny Set	3_1_2	3	0	44.7518	0.9681	0.0319	0.0164	0.0371	-540.9835	-426.8844	94	0.0079	1_2
	3_1_2	1	0	0.3123	0.7750	0.2250	0.0170	0.0177	-1093.0502	-1105.8170	183	0.1080	3_2
	3_1_2	2	0	42.2040	0.9904	0.0096	0.0136	0.0191	-659.8777	-610.9846	104	0.0026	3_1

Note for headline:

Outgroup: *T. yunnanensis*; 1: *M. glyptostroboides*; 2: *S. giganteum*; 3: *S. sempervirens*.

C1, C2: The time (in coalescent units) since two sister species became isolated from the third species for a triplet topology under the ILS-only and the ILS+introgression distribution model, respectively.

mixprop1, mixprop2: The inferred mixing proroptions for the ILS-only and ILS+introgression distribution model, respectively;

lambda2Dist, lambda1Dist: The scaling factor to convert the input branch length unit into coalescent units for the ILS+introgression and the ILS-only distribution model, respectively.

BIC1Dist, BIC2Dist: The Bayesian Information Criterion scores for the ILS-only and ILS+introgression model, respectively;

Count: The total number of trees supporting a triplet topology.

totalIntroProp: Proportion of trees arising via introgression.

Table S14. Descriptions and accession numbers of sequencing data.

Database	Project	Sample/Run ID	Sample Name	Type	Tissue
M. glyptostroboides					
CNCB	PRJCA016596	SAMC1221263	Mgly_genome	genome assembly	leaf
CNGBdb	CNP0003114	CNX0479691	Mgly_hic	HiC/Nova	leaf
CNGBdb	CNP0003114	CNX0479669	Nova_genomic	WGS	leaf
CNGBdb	CNP0003114	CNX0465137	ONT_genomic	ONT genomic	leaf
CNGBdb	CNP0004335	CNS0773882	ONT_cDNA	ONT cDNA	multiple tissues
CNGBdb	CNP0004335	CNS0773883	Camb1	RNA-seq	cambium
CNGBdb	CNP0004335	CNS0773884	Camb2	RNA-seq	cambium
CNGBdb	CNP0004335	CNS0773885	Camb3	RNA-seq	cambium
CNGBdb	CNP0004335	CNS0773886	MR0-1	RNA-seq	root
CNGBdb	CNP0004335	CNS0773887	MR0-2	RNA-seq	root
CNGBdb	CNP0004335	CNS0773888	MR0-3	RNA-seq	root
CNGBdb	CNP0004335	CNS0773889	MR3-1	RNA-seq	root
CNGBdb	CNP0004335	CNS0773890	MR3-2	RNA-seq	root
CNGBdb	CNP0004335	CNS0773891	MR3-3	RNA-seq	root
CNGBdb	CNP0004335	CNS0773892	MR6-1	RNA-seq	root
CNGBdb	CNP0004335	CNS0773893	MR6-2	RNA-seq	root
CNGBdb	CNP0004335	CNS0773894	MR6-3	RNA-seq	root
CNGBdb	CNP0004335	CNS0773895	MR9-1	RNA-seq	root
CNGBdb	CNP0004335	CNS0773896	MR9-2	RNA-seq	root
CNGBdb	CNP0004335	CNS0773897	MR9-3	RNA-seq	root
CNGBdb	CNP0004335	CNS0773898	MR12-1	RNA-seq	root
CNGBdb	CNP0004335	CNS0773899	MR12-2	RNA-seq	root
CNGBdb	CNP0004335	CNS0773900	MR12-3	RNA-seq	root
CNGBdb	CNP0004335	CNS0773901	YB1	RNA-seq	bud
CNGBdb	CNP0004335	CNS0773902	YB2	RNA-seq	bud
CNGBdb	CNP0004335	CNS0773903	YB3	RNA-seq	bud
CNGBdb	CNP0004335	CNS0773904	YS-1	RNA-seq	stem
CNGBdb	CNP0004335	CNS0773905	YS-2	RNA-seq	stem
CNGBdb	CNP0004335	CNS0773906	YS-3	RNA-seq	stem
S. sempervirens					
CNGBdb	CNP0004335	CNS0798239	Ssem_genome	genome assembly	leaf
CNGBdb	CNP0004335	CNS0773907	cell1	HiFi	leaf
CNGBdb	CNP0004335	CNS0773908	cell2	HiFi	leaf
CNGBdb	CNP0004335	CNS0773909	cell3	HiFi	leaf
CNGBdb	CNP0004335	CNS0773910	cell4	HiFi	leaf
